# Health Promotion in Early-Stage Dementia: A Focused Ethnographic Study of a 12-Week Group-Based Educational Intervention

**DOI:** 10.1177/23779608241266686

**Published:** 2024-07-25

**Authors:** Martine Kajander, Martha Therese Gjestsen, Clive Ballard, Halvor Næss, Ingelin Testad

**Affiliations:** 1 Centre for Age-Related Medicine - SESAM, 60496 Stavanger University Hospital, Stavanger, Norway; 2 1658 University of Bergen, Department of Clinical Medicine, Bergen, Norway; 3 3286 University of Exeter, Faculty of Health and Life Sciences, Exeter, UK; 4 Haukeland University Hospital, Department of Neurology, Bergen, Norway

**Keywords:** dementia, home-dwelling, health promotion, education, support, focused ethnography

## Abstract

**Introduction:**

Educational health promotion interventions for people with early-stage dementia have shown promising results, including empowering the person with dementia to live well and cope with their condition.

**Objective(s):**

The aim of this study was to explore how group interactions, course structure, and facilitation by healthcare professionals in a 12-week educational health promotion course promote coping, healthy behaviors, and empowerment in people with early-stage dementia.

**Method:**

A focused ethnographic approach was employed, collecting data through moderate participant observations of people with early-stage dementia who attended the health promotion course and field conversations with the facilitators. Additionally, before and after the participants had completed the course, the participants and their care partners were interviewed individually.

**Results:**

The findings showed that group discussions provided an opportunity for the facilitators to identify knowledge gaps, correct misinterpretations of symptoms, and tailor the information to the participants’ specific needs, thereby promoting healthy behaviors and empowering the participants. The consistent and structured format of the course appeared to reduce stress and promote learning. Learning about dementia first-hand, reminiscing, using humor, receiving support from others facing similar challenges, and receiving support and validation from facilitators all contributed to participants coping with their condition, processing negative emotions, and reducing internalized stigma.

**Conclusion:**

This study emphasized the importance of providing people living with early-stage dementia educational opportunities that combine first-hand information, peer and facilitator support, reminiscing, humor, recognition, and validation. These interventions can contribute to promote coping, healthy behaviors, and empowerment in people living with early-stage dementia.

## Introduction

A diagnosis of dementia fundamentally changes a person's life. Following this diagnosis, people living with dementia are particularly vulnerable, and many experience a variety of emotional reactions (Górska et al., 2018) in addition to being subjected to dementia-related stigma (O'Connor et al., 2018). Lack of information and support in the early stages of the condition can lead to social isolation and difficulties adjusting to and coping with the new situation (Górska et al., 2018; Norwegian Ministry of Health and Care Services, 2015). In the absence of a cure or highly effective treatment, educational interventions such as group-based health promotion interventions for people with early-stage dementia have gained increased attention (Dal Bello-Haas et al., 2014; Daly et al., 2022). Educational health promotion interventions have the potential to help individuals with dementia to better adapt to and cope with their condition by providing first-hand knowledge about their diagnosis (Daly et al., 2022). In addition, by focusing on establishing healthy behaviors during the condition's early stages, many avoidable secondary conditions related to dementia (depressive symptoms, injuries, falls, and nutritional issues) can be reduced or prevented, which, in turn, can contribute to preventing premature institutionalization (Buettner & Fitzsimmons, 2009; Manthorpe & Moniz-Cook, 2020).

## Review of Literature

Research on educational health promotion and similar interventions, such as self-management interventions for people living with early-stage dementia have been promising, however, the evaluation and implementation of these interventions in clinical practice remains scarce (Keogh et al., 2019; Quinn et al., 2015). A review by Keogh et al. (2019) found that only 5 out of 69 intervention studies were group-based educational health promotion and self-management interventions. The remaining were cognitive-oriented, psychotherapeutic, support groups, multicomponent, or physical activity interventions. These interventions share elements with educational health promotion interventions for people with dementia, such as providing information about dementia and peer support (Leung et al., 2015; Morton et al., 2021). However, educational health promotion interventions differ from other interventions, with a structured educational approach placing a strong emphasis on integrating healthy behaviors into daily life and developing coping skills during the early stages of dementia.

The educational health promotion and similar interventions included in Keogh et al. (2019) showed significant positive changes in cognition (Laakkonen et al., 2016) and improved self-efficacy (Quinn et al., 2016; Richeson et al., 2007; Sprange et al., 2015). The studies also reported that participants valued the social aspects of the intervention and the opportunity to learn about dementia (Fitzsimmons & Buettner, 2003; Quinn et al., 2016; Richeson et al., 2007; Sprange et al., 2015). Studies not included in the review or published afterward have shown significant positive changes in cognition, improvement in depressive symptoms (Buettner & Fitzsimmons, 2009), and better adjustment to and coping with the condition (Sprange et al., 2021). Studies have also found that participation contributed to increased openness and acceptance of the diagnosis and improved family communication (Øksnebjerg et al., 2020; Sims & McCrum, 2012). Although most studies have reported positive findings, a recent randomized controlled trial by Mountain et al. (2022) on a self-management intervention for individuals with mild dementia concluded that the intervention was neither clinically effective nor cost-effective. The authors suggest that existing measures may not be sensitive enough to capture meaningful changes in interventions for people with mild dementia (Mountain et al., 2022). However, they did note improved psychological well-being in the intervention group, which was supported by positive qualitative outcomes, as reported by the same group (Sprange et al., 2015, 2021).

The existing reports about participants’ experiences of these interventions and the impact of the interventions offer valuable perspectives. However, there remains a paucity of qualitative research in this field (Keogh et al., 2019), and studies on educational health promotion interventions employing direct observations are absent. Thus, there is very limited knowledge how the internal dynamics and processes within these groups contribute to the positive outcomes reported by participants. More qualitative research is essential to document and explore the internal dynamics and processes within these groups, which can offer important insight into what makes educational health promotion interventions for people living with early-stage dementia “work” (Dugmore et al., 2015). Gaining a deeper understanding of these elements is essential for developing and optimizing future interventions to meet the unique needs of individuals living with early-stage dementia.

This study employed a focused ethnographic approach using moderate participant observations. The aim was to explore how group interactions, the course structure, and the facilitators in a 12-week health promotion course contributed to promoting coping, healthy behaviors, and empowerment in people with early-stage dementia. Directly observing the process inside these groups can help inform government initiatives aimed at educating and supporting people with early-stage dementia.

## Method

### Study Design

This study was part of a larger project entitled “*Health promotion for persons with dementia; Prevention of hospitalisation and nursing home replacement for persons with dementia,*” which included a 12-week health course for people with early-stage dementia (Testad et al., 2020). The overall aim was to provide information about dementia and the course of the condition, addressing preventable measures and supporting the development of healthy behaviors to help prevent problems that can occur in the later stages of the condition and thereby prevent premature institutionalization (Testad et al., 2020). The intervention demonstrated a significant improvement in depressive symptoms and self-rated health in people with early-stage dementia (Testad et al., 2020). As part of this project, a qualitative interview study was also conducted, it showed that the intervention improved coping, promoted healthy behaviors, and empowered the person with dementia (Kajander et al., 2022).

This study employed a focused ethnographic approach including moderate participant observations and semistructured interviews (Higginbottom et al., 2013). Focused ethnography is an adapted form of ethnography that enables the exploration of a specific context or setting, involves a limited number of participants who are purposefully sampled, and entails concentrated field trips over a relatively limited time frame (Andreassen et al., 2020; Higginbottom et al., 2013). The approach was deemed appropriate for this study because it allowed for an in-depth exploration of a specific setting with specific characteristics (i.e., the health promotion course) through observation of behaviors, social interactions, and group dynamics (Andreassen et al., 2020; Knoblauch, 2005).

### Intervention

The intervention was group-based and consisted of 12 weekly two-hour sessions. During the first session, all participants received a course-specific booklet that covered the course content (Table 1). All sessions followed a structured format to provide consistency and familiarity. Each session began with a breathing exercise, followed by the lead facilitator introducing the day's topic and presenting the main points through a discussion with the participants. Halfway through the session, a short break was offered and refreshments were served. Each session finished with goal setting.

**Table 1.** Topics Covered in the 12-Week Educational Health Promotion Course.

Session	Title of the session
1	Healthy lifestyles
2	Dementia and delirium
3	Cognitive activities
4	Communication and memory
5	Coping in dementia
6	Physical activity
7	Home and travel safety
8	Recreation and leisure
9	Lifelong learning
10	Medications and talking to your healthcare provider
11	Nutrition
12	Future planning

*Note*: Adapted from “*Health promotion intervention for people with early-stage dementia: A quasi-experimental study*“ by Testad et al. (2020, p. 2), published under a CC BY 4.0 license.

Each group consisted of up to seven participants and was facilitated by two registered nurses with longstanding clinical experience in caring for older persons. To ensure quality and consistency, the facilitators received comprehensive training before delivering the intervention and followed a manual outlining the main elements, structure, and time allotments for the content of each session. Facilitators also received guidance from the registered nurse, who holds a PhD in care and nonpharmacological interventions for people with dementia and is the professor who developed the intervention based on a health promotion course for people with early-stage dementia developed by Fitzsimmons and Buettner (2003). The purpose of guidance (provided following the third, seventh, and eleventh sessions) was to give all facilitators the same support and information aligned with the overall aim of the educational health promotion intervention. The conceptual framework of the intervention is based on the Corbin and Strauss Chronic Illness Trajectory Model (Corbin & Strauss, 1991). The model is founded on the premise that chronic conditions, such as dementia, can be managed and shaped over time, even if the condition's natural progression cannot be modified (Corbin & Strauss, 1991). Central to the model is health promotion, with the aim of preventing or reducing the impact of problems that may arise in the later stages of the condition (Corbin & Strauss, 1991).

### Research Questions

The following research questions guided the study:

- How can interacting with other group members promote coping, healthy behaviors, and empower people with early-stage dementia?
- How can the course structure promote coping, healthy behaviors, and empower people with early-stage dementia?
- How can group facilitators promote coping, healthy behaviors, and empower people with early-stage dementia?

### Recruitment and Sample

The main project ran from 2014 to 2019 and delivered 26 health promotion courses in urban and rural parts of Norway's western, eastern, and northern regions (Testad et al., 2020). Participants were recruited from primary care, general practitioners’ offices, memory clinics, daycare centers, and newspaper advertisements. This study used a convenience sample and included all 12 sessions of the two health promotion groups held in Western Norway between September 2018 and February 2019.

### Inclusion and Exclusion Criteria

The primary inclusion criteria were home-dwelling individuals aged 65 or older, in the early or moderate stages of dementia (i.e., a Clinical Dementia Rating score ≥2 [Morris, 1993]), diagnosed with Alzheimer's or vascular dementia, capable of reading and writing, hearing and vision sufficiently good to function in a group setting, proficient in Norwegian, and the capacity to provide informed consent. A research nurse assessed participants’ capacities to provide informed consent. The exclusion criteria were a diagnosis of alcohol abuse, limited life expectancy, ongoing chemotherapy, or radiation treatment at date of enrollment, head injury, epilepsy, Parkinson's disease, a history of psychiatric illness or diagnosis of subnormal intelligence, prior participation in health promotion or cognitive training programs (Testad et al., 2020).

## Data Collection

The data were collected through moderate participant observations and individual interviews.

### Observations

The 12-week educational sessions took place at a local community-based activity center in a private and quiet room, with chairs arranged around a square table to encourage conversation among group members. In this study, moderate participant observation was conducted (DeWalt & DeWalt, 2011), as this enables the researcher to be present in the room with the participants and at the same time focus, on writing field notes and capturing verbatim quotes during the sessions. Although the observer is sitting on a chair in the back of the room and is not an active participant in the group setting, moderate participant observation allows the observer to occasionally interact with the participants (for example, if participants ask questions or when dictated by common courtesy, such as greetings at the start and end of each session). This contributes to integrating the observer as a natural part of the group setting, reducing the observer's impact on the participants (DeWalt & DeWalt, 2011). Observations were guided by a semistructured observation guide building on the findings from a qualitative study by Kajander et al. (2022). The first author conducted all observations and had met the facilitators during other phases of the project, which contributed to easing the observer's presence during intervention delivery. Directly following each session, the observer and facilitators sat down together to debrief and discuss their preliminary impressions of the session. Descriptive field notes were taken during the sessions (see Supplemental file 1 for an example). Immediately after each session, the field notes and the researchers’ reflections on the observed session were transcribed into detailed and coherent descriptions (Emerson et al., 1995). The cumulative time in the field was 72 h, which included the 30 min the researcher spent before the sessions started and the 30 min used for debriefing and discussion with the facilitators.

### Interviews

Individual interviews with participants and their care partners were conducted before and following (within two weeks of) the 12-week course. Participants and their care partners were asked the same questions (Supplemental file 2). The participants (the person living with dementia) were the main informants, whereas the care partner interviews provided a complementary perspective based on their daily interactions with the participants over the 12 weeks. The interviews took place at a university hospital and lasted approximately 60 min. The first and last authors conducted the interviews, which were recorded through handwritten notes and transcribed immediately afterward.

The concept of information power, which is related to saturation, was used to evaluate the sufficiency of information derived from the observations and interviews (Malterud et al., 2016). The information power was enhanced through multiple data sources (Malterud et al., 2016), and it was considered sufficient after observations of all the sessions of the second group including participant and care partner interviews. No new themes emerged, and the data material contained (taking into account the nature of the participants’ condition, involving language and memory impairments) a clear dialogue and thorough descriptions (Malterud et al., 2016).

## Ethical Consideration

The study was conducted in accordance with the Helsinki Declaration (World Medical Association, 2013) and approved by the Regional Committees for Medical and Health Research Ethics, REC North (2013/2266). Written informed consent was obtained before the interviews and observations. At the start of each session, information about the observations, confidentiality, and anonymity of the data collected was repeated. The study is registered with ClinicalTrials.gov (NCT03741543).

## Analysis

The data were analyzed using content analysis as outlined by Graneheim and Lundman (2004), which aims to describe the “manifest” and “latent” contents of the material—in other words, what the text explicitly states (manifest) and the underlying meaning of what is being said (latent) during observations and interviews. Data derived from the moderate participant observations, individual interviews with participants and care partners, and field conversations with the facilitators were combined through a continuous cyclic process to facilitate triangulation (Higginbottom et al., 2013). The analysis comprised the following steps:

1. The first and last authors read through the transcripts multiple times to gain a sense of the whole.
2. Meaning units related to the research question were identified and each meaning unit was condensed into a shorter text describing the content and underlying meaning. To ensure the trustworthiness of the analysis, a discussion about the meaning units, including manifest and latent content, was held with an interdisciplinary group of researchers with backgrounds in social sciences, theology, and nursing.
3. Subthemes were then created through an abstraction of descriptions and underlying meaning of the meaning units. During this stage of the analysis, the preliminary findings were presented to the project's user group to discuss alternative interpretations and reflect on the subthemes. The user group was formed to ensure patient and public involvement in this study and included a person with dementia, a care partner, a representative from the Norwegian Health Association, and one retired nurse.
4. After re-organizing subthemes based on the user group's input, and moving back and forth between the whole and parts of the text, consensus on the final themes and subthemes was achieved by the authors. Table 2 shows a simplified example of the analysis.

**Table 2.** A Simplified Example of the Analysis.

Meaning units (selected)	Condensed meaning unit (manifest)	Condensed meaning unit (latent)	Subtheme	Main theme
Participant 1107: *I don't feel like I'm ever hungry anymore. I may even forget to eat at times. My wife is constantly reminding me. [….]*	Don’t feel hungry, needs to be reminded	Unaware not feeling hungry is a symptom of dementia	Utilizing group discussions to identify knowledge gaps	Empowered through increased knowledge and mutual support
Participant 1101: *I drink juice, that is quite healthy.* Participant 1102: *I drink… Oh what was the name again…* Participant 1100: *Milk?* Participant 1103: *Hm, could it be…* Participant 1102: *Oh, I have it on the tip of my tongue, give me a second…* [the other participants remained silent] *Kefir… Yes!*	Two group members helped a third member of the group remember the name of a beverage	Not having to worry about finding the right words	Promoting social participation through social stimuli, facilitation, and course structure	

*Note*: A meaning unit is a segment of text that contains relevant information related to the research question.

### Preunderstanding

The first, second, and last authors were all familiar with the study context from collecting and analyzing data on prior health promotion groups. The third and fourth authors’ preunderstandings were related to their clinical and research experience within geriatric psychiatry and neurology. The last author was responsible for developing the intervention. None of the authors were involved in delivering the intervention.

## Results

### Participant Characteristics

Two health promotion groups were selected for observations, group A comprised four participants (2 males, 2 females) and group B comprised five participants (2 males, 3 females). In addition, the participants and their care partners were interviewed before and following the 12-week intervention. Participant characteristics are summarized in Table 3.

**Table 3.** Sociodemographic Characteristics of the Sample.

Selected variables		Person with dementia (*n* = 9)	Care partner (*n* = 9)
Sex			
	Male	4 (44%)	5 (56%)
	Female	5 (56%)	4 (44%)
Age, mean (range)		75 (68–86)	69 (46–85)
Marital status (person with dementia)			
	Married	7 (78%)	
	Widowed	1 (11%)	
	Divorced/separated	1 (11%)	
Living situation			
	Living with spouse	7 (78%)	
	Living alone	2 (22%)	
Clinical Dementia Rating Scale Score			
	0.5 very mild dementia	6 (67%)	
	1 mild dementia	3 (33%)	
The care partner's relationship with the person with dementia			
	Spouse		7 (78%)
	Daughter/son/grandchild		2 (22%)

*Note*: The data are reported as counts and percentages unless otherwise specified.

### Research Findings

The data analysis resulted in two main themes: (1) empowerment through increased knowledge and mutual support, and (2) a respectful and nonjudgmental group environment. Theme 1 addresses all three research questions (interacting with other group members, the course structure, and how the facilitators promote coping, health, and empowerment in people with early-stage dementia). Theme 2 primarily addresses the first and third research questions. Within the main themes, four subthemes were identified (Table 4).

**Table 4.** Main Themes and Subthemes.

Main themes	Subthemes
Empowered through increased knowledge and mutual support	Utilizing group discussions to identify knowledge gaps Promoting social participation through shared experiences, facilitation, and course structure
A respectful and nonjudgmental group environment	Recognition and understanding versus belittlement and criticism Using humor as a buffer against dementia-related stigma

## Main Theme 1: Empowered Through Increased Knowledge and Mutual Support

The group discussions, guided by each session's topic, provided an opportunity for facilitators to identify the participants’ knowledge gaps, correct misinterpretations of symptoms, and adjust information to meet specific needs. Through facing similar challenges of living with dementia and being able to contribute, the participants felt a sense of community and usefulness to others. The course structure, facilitators, and mutual support appeared to compensate for the participants’ language impairments and promoted social participation.

## Utilizing Group Discussions to Identify Knowledge Gaps

The level of dementia knowledge differed among the participants in the two groups. It was apparent from observing group discussions that several participants were unaware of many of the symptoms and characteristics of the condition. Through group discussions, the facilitators were able to link experiences in daily life, such as loss of appetite, to dementia symptoms.

Participant 1107: *I don't feel like I'm ever hungry anymore. I may even forget to eat at times. My wife is constantly reminding me.*

Facilitator: *Do you feel hungry when you start eating?*

Participant 1107: *Yes, when I start eating, it feels good because I’m hungry. It just never occurs to me. I used to fuss about the next meal all the time. Now my wife tells me I never ask for food.*

Facilitator: *Not feeling hungry is a symptom of dementia, it is important to establish a mealtime routine.*

Participant 1107: *Really? I must tell my wife!*

(Field notes Group B)

The group discussions also revealed that several participants misinterpreted some of the symptoms of dementia, i.e., misplacing objects or becoming passive, and viewed these as personality traits. Others tried to rationalize their symptoms by referring to memory problems as a normal part of aging.

Participant 1101: *It's important to tell people that as you age, you become forgetful. There's nothing to be concerned about.*

Facilitator: *However, dementia is not a normal part of ageing.*

(Field notes Group A)

Dementia and related symptoms were talked about in a courteous yet clear and direct manner by the facilitators without softening the unpleasant truths of the condition. The facilitators utilized misinterpretations of symptoms to help participants sort out and objectively define these challenges as condition-related, not personal traits. For example, misplacing objects or having difficulty finding the right words was changed from a personal defeat to a problem related to dementia. This appeared to empower the participants and provide renewed confidence in coping with their condition. Two care partners stated:

Care partner 2107: *He has gone from feeling useless to being a competent person who happens to have Alzheimer's disease.*

Care partner 2108: *He's more confident now and doesn’t believe his life is coming to an end. Now we can make plans for the future, which he previously refused to do. He has truly benefited from taking the course. He has started to see his condition in a new light.*

### Promoting Social Participation Through Shared Experiences, Facilitation, and Course Structure

During group discussions, several participants talked about their language impairments that created barriers to initiating and engaging in conversations. However, within the group, these challenges received little attention, as participants focused on listening to and supporting one another. Occasionally the whole group got involved in assisting another group member in recalling a specific name or place:

Participant 1100: *Recently, I was at a concert at a restaurant… oh what was the name…*

Participant 1102: Was it Joe's*?

Participant 1100: No, not Joe's*.

Participant 1101: *Hmm… What street was it in?*

Participant 1100: Ahhh! It was at Eternity*. I went there with my daughter. Her husband was working, so she invited me.

(Field notes Group A. *Restaurant names are fictive)

The observations also recorded that facilitators used reminiscence to encourage conversations. Talking about the past and relating the course material to the participants’ lives when they were younger appeared to facilitate conversations in which the participants could speak with confidence to others who were interested in listening:

[…]

Participant 1105: *My parents lost their farm during World War II. They eventually got it back, but they were still required to live in their parents’ house. I recall seeing Polish prisoners in the house next door…*

Participant 1106: …*and there were Germans everywhere.*

Participant 1104: *I remember the Germans constantly asked for “bom-bom”* [sweets] *because it was the only Norwegian word they knew.* [laughs]

(Field notes Group B)

Being in a group in which other participants were interested in what someone had to say, and understood what they were going through, appeared to make it easier to talk without worrying about finding the right words, being repetitive, or speaking slowly.

Participant 1107*: Before I retired, I used to travel around and give presentations. I could never do that now.*

Participant 1106*: But no one expects you to do that.*

Participant 1107*: That's true, although it's much easier to talk here because you all understand what I'm struggling with.*

(Field notes Group B)

Other participants emphasized that the educational structure of the course had made it easier to talk within the group. The consistent structure, where a new topic was presented each session and the relevance of the topics to their life situation, ensured that the conversations did not rely on any given participant's ability to come up with a suitable topic to discuss.

Participant 1101: *It was nice to meet new people and learn and to talk about a variety of topics. It felt good. I don’t usually say much, and even I haven’t been afraid of speaking up. It was not too theoretical, and that was good. It was focused on what we can do, on helping each other.*

Being in a group with peers also appeared to give the participants a sense of community. The participants were observed helping one another locate the correct page in the booklet, and expressing concern if a fellow group member missed a session or was running late. By being able to contribute, the participants felt useful to others.

Care partner 2106: *She felt she had an opportunity to give something back to the group by encouraging those who were silent to speak up. Too many people are embarrassed by their problems. She was delighted to contribute.*

The observations further recorded that as the sessions progressed, several participants appeared to be more confident about talking, sharing, and asking questions, which empowered some participants to stop withdrawing from conversations outside of the group.

Participant 1107: *The course helped me learn that I can still participate, although it's a bit difficult, and I am slow-paced. I used to talk up a storm. Now it's more like a whistling wind.*

### Main Theme 2: A Respectful and Nonjudgmental Group Environment

The supportive environment within the group appeared to be quite different from the participants’ daily lives outside of the group. In contrast to being belittled or criticized, the participants’ difficulties were taken seriously, there was no judgment and they were heard, which appeared to offer a safe space to vent their frustrations. The group environment also allowed for humor and irony, which appeared to lighten the group's mood during discussions about the general public's perception of dementia.

### Recognition and Understanding Versus Belittlement and Criticism

The respectful and supportive nature of the group appeared to foster an open dialogue among group members and between group members and facilitators. Several participants reported being surrounded by equals who took each other seriously, which made sharing difficulties easier.

Participant 1107: *Another thing I noticed was that we were all equals, with no one superior or inferior to anyone else. We took each other seriously*, *I wasn't afraid to say things because I wasn't afraid of looking stupid.*

The facilitators showed genuine interest in the participants’ lives, treated them respectfully, and displayed nonjudgmental attitudes.

Participant 1104: *There were no stupid questions. The woman* [the facilitator] *on the other side of the table was not “above” us. There was no top-down attitude. She didn’t ask questions because she was curious; she asked because she wanted to understand.*

Being met with respect and genuine interest appeared to have contributed to the group meetings becoming a safe space, allowing participants to talk freely about challenging aspects of their lives and vent their frustrations. During the group discussions, participants talked about their spouses or children occasionally showing frustration about their memory difficulties, made hurtful remarks, or criticized them if they had forgotten something.

Participant 1107*: My wife will occasionally ask, “don't you remember this?” or “did you forget that?” It's insensitive.*

Participant 1106: *I know! “Don't you remember that?” It's how they say it.*

(Field notes Group B)

Other participants felt that their friends did not take their condition seriously and could downplay their symptoms by saying things like “we also forget,” or “you should just try to eat this or that,” causing distress and frustration. Consequently, some participants began to withdraw from the people around them.

Participant 1103*: I get offended when other people don’t understand what it's like to have dementia. It's a shame. It's due to their ignorance. It makes me feel uncomfortable. It hurts as well.*

(Field notes Group A)

The facilitators were observed validating the participants’ experiences and, when appropriate, encouraging participants to consider alternative explanations for other people's behaviors. An excerpt from one of several conversations between a participant and a facilitator illustrates this:

Participant 1103*: The people around me don’t understand; they tell me that they experience the same things as me and that they also forget. But it's not the same.*

Facilitator: *Your friends saying that they also forget is not the same as having dementia. Could it perhaps be that they are trying to console you? Or maybe they simply don’t know what to say?*

(Field notes Group A)

As the sessions progressed, this validation further appeared to encourage one of the participants to not give up on a complicated matter, and shared this determination with the participants:

Participant 1103*: I’ve spoken with my friend. She was the one who called me. But it was nice.*

Facilitator: *That's good!*

Participant 1103*: My next step should be to pay her a surprise visit.*

(Field notes Group A)

Many participants repeatedly talked about the same issues during the 12-week course, but as the weeks went by, they described those issues with increasingly less frustration. One of the participants was in the process of losing his driving license and shared this with the group. The other participants expressed sympathy for this loss and shared their stories about losing their driving privileges. As the weeks went by, the observations noted the participant adding some humor to the situation:

Participant 1108*: Yeah, walking distance is all I think about now.*

(Field notes Group B)

### Using Humor as a Buffer Against Dementia-Related Stigma

The group meetings further appeared to be a place where participants could share and discuss their concerns about the general public's perception of dementia. Most of the participants had chosen to be open about their condition. However, they often encountered uninformed views from the people around them and described the general public as insensitive and poorly informed.

Participant 1106: *There are many strange opinions out there. I told a few friends I hadn’t seen in a while* [that I have been diagnosed with dementia]*. They later told my husband that they thought I was perfectly normal. It's laughable. What were they thinking? That I'd turn into a troll?*

(Field notes Group B)

The participants also discussed the negative connotations associated with dementia, which had made some of them reluctant to use the word at all.

Participant 1103: *I know I have dementia, but I don’t…*

Participant 1101*: No, I don't use such a sad word like dementia because it makes people think I'm insane and that they can't rely on me. I don't want to make things any worse than they are.*

Participant 1103: *There's something negative attached to that word, but it's the reason I behave the way I do. When most people hear the word dementia, they just picture someone acting out.*

(Field notes Group A)

Other participants appeared to spend a lot of energy trying to educate the people around them, feeling compelled to defend themselves against the public's perceptions of dementia:

Participant 1103: *I figured, if I don’t come forward myself, people will start making assumptions about me being a drunk because of the way I behave. Because people are so ignorant, I feel like I have to inform them.*

(Field notes Group A)

Although the course focused on living with dementia and group discussions centered on serious subjects, some participants were observed using humor and irony, for example when talking about their own cognitive challenges or responding to another group member's challenges or concerns. In addition to learning about their condition and sharing experiences with peers and supportive facilitators, it appeared that being able to laugh also helped empower the participants. Humor and irony appeared to alleviate tension, lift the participants’ spirits, and act as a buffer against their concerns about the public's perceptions of dementia:

Participant 1103: *After getting diagnosed, I wanted my neighbours to understand if my behaviour changed, in case I got confused and started getting up and wandering around at night.*

Participant 1100: *If you start wandering around at night and they notice it, just tell them you've become a Night Raven* [a voluntary citizen night-time patrol group]. [laughs] *That will keep them quiet.*

Participant 1103: *I better remember that.* [laughs]

(Field notes Group A)

Several participants emphasized the importance of being able to make jokes and laugh, and the fact that the course structure was not too serious:

Participant 1101: *A lovely atmosphere and a lot of humour. It was very refreshing to be with the other members of the group.*

One care partner also reported:

Care partner 2102: *She enjoyed herself, talking to the others. She mentioned that one of the group members was quite witty.*

## Discussion

This study explored how interactions among participants, the course structure, and the facilitators contributed to coping, health promotion, and empowerment in people with early-stage dementia who attended a 12-week course. Directly observing the groups revealed that through group discussions, facilitators were able to identify knowledge gaps, correct misinterpretations of symptoms, and tailor information to meet specific needs, thereby promoting healthy behaviors and empowering the participants. Learning about dementia first-hand, the support and recognition of others facing similar challenges, reminiscing and using humor, as well as the support and validation of the facilitators, helped the participants process negative emotions, empowered them to cope with different symptoms, and reduced internalized stigma.

Previous research has highlighted that individuals with dementia and their care partners often receive limited or inadequate information regarding the condition and its associated symptoms (Frost et al., 2020; Wheatley et al., 2021). The present study's findings support previous research, and as revealed in the group discussions, the participants frequently misinterpreted their symptoms and lacked a basic understanding of key aspects of health promotion in dementia. Previous research has also noted that many patients face difficulties comprehending and utilizing the oral and written information provided by healthcare services (Murugesu et al., 2022) and may not necessarily know what questions to ask (Innes et al., 2014).

Another finding was that the consistent and structured format of the course, with the introduction of a new topic in each session and subsequent group discussions focused on these subjects, appeared to reduce the participants’ stress because they were not responsible for coming up with conversational topics. According to Hall and Buckwalter (1987), people with dementia are especially vulnerable to internal and external stressors. Stress is known to impair cognitive functioning (Hall & Buckwalter, 1987); therefore, lowering the participants’ stress is crucial when the aim is active group participation and improving the participants’ knowledge about dementia and related symptoms.

In line with findings from similar group-based interventions (Martin et al., 2015; Quinn et al., 2016) and support groups (Hedman et al., 2014), the support process and interactions among participants fostered a sense of community, where the participants’ contributions were valued, and they felt useful to others. This appeared to empower some of the participants to cope with their language impairments. The social interactions the participants talked about encountering outside of the group, on the other hand, appear to undermine their personhood (Kitwood, 1997), making them feel belittled, devalued, and mocked. These negative social interactions between a person with dementia and a person close to them have rarely been reported in the research literature (Mazaheri et al., 2013). However, these types of social interactions are known to disempower people with dementia and can lead to social withdrawal (Birt et al., 2020). For some of the participants, the group meetings appeared to provide a very important space that might not otherwise exist in their lives, where they got the opportunity to reminisce, talk freely, and feel heard. These findings are in line with findings from support groups for people living with dementia (Ward et al., 2012) and could have contributed to helping participants work through some of their emotional distress, as some of the participants were observed talking about challenges and worries with less frustration over the duration of the course.

The findings indicated that involving healthcare professionals in group facilitation was essential because of the knowledge and skills needed to support the participants’ comprehension of the complex nature of dementia. Knowledge beyond a basic understanding of dementia appears to be necessary to help participants recognize challenges originating from dementia and distinguish these from personal traits. In this study, challenges and misinterpretations of symptoms indirectly surfaced during group discussions. Furthermore, the facilitators’ role also involved challenging the participants’ mindsets and encouraging participants to consider alternative explanations for other people's behaviors. Thus, active problem-solving and an understanding of the situation were emphasized rather than merely sharing difficulties. For example, because of the support, validation, and encouragement of the facilitators, one participant felt empowered to give a complicated friendship a second chance.

It appeared that participants experienced stigma and had internalized negative stereotypes associated with dementia. This internalization of public stigma is in line with previous research exploring the experience of living with dementia in the community (O’Sullivan et al., 2014). Stigma is known to discourage people with dementia and their families from seeking care and support from healthcare services (Martin et al., 2013; Werner et al., 2014), which increases the risk of numerous preventable secondary conditions going unnoticed and untreated (Norwegian Ministry of Health and Care Services, 2015). The findings indicate that the intervention contributed to reducing participants’ internalized stigma by drawing on the two main strategies to combat stigma, namely education and meeting others with the same condition (identified in studies on other stigmatizing conditions, Corrigan & Penn, 1999). Most strategies designed to combat dementia-related stigma aim to educate the public and thereby decrease self-stigma (Kim et al., 2021). This study showed that group discussions played an important role in reducing stigma by specifically addressing the persons affected, identifying and correcting misinterpretation of symptoms, and helping them replace inaccurate stereotypes with factual, research-based information about the condition.

Another interesting observation was that the participants’ use of humor when talking about stigmatization, helped them express their experiences and worries about being stigmatized. Bjørkløf and colleagues (2019) found that people with dementia use self-directed humor to cope with cognitive changes; viewing their cognitive difficulties in a more light-hearted manner helps reduce the condition's impact on their lives. In general, though, the use of humor in educational health promotion and similar interventions has received little attention, particularly in the context of dementia-related stigma reduction.

## Implications for Practice

The findings stress the importance of considering how information is delivered to people with early-stage dementia. Traditional information provision may fall short, as people may not necessarily know what to ask and misinterpret symptoms. Moreover, the participants’ unfamiliarity with secondary symptoms like reduced appetite poses significant health risks, as people with dementia are particularly vulnerable to malnutrition and weight loss (Volkert et al., 2019), which is associated with accelerated cognitive decline (Sanders et al., 2016), functional decline, and higher mortality risk (Borda et al., 2021). These findings emphasize the importance of ensuring that facilitators delivering these interventions have knowledge extending beyond a basic understanding of dementia to help participants recognize challenges originating from dementia, distinguish these from personal traits, and, most importantly, help them find ways to cope with these symptoms.

## Strengths and Limitations

Possible researcher bias needs to be considered, as only one researcher, with an insider perspective, conducted the fieldwork. Overfamiliarity can lead to significant and novel aspects being overlooked (Polit & Beck, 2017). At the same time, because focused ethnography involves short-term field visits, familiarity with the context and the participants’ terminology can be an advantage (Higginbottom et al., 2013; Knoblauch, 2005). Several strategies were employed to enhance the study's trustworthiness, using the criteria outlined by Lincoln and Guba (1985). The key strength of this study, which contributed to ensuring credibility and reducing researcher bias, was the focused ethnographic approach and the triangulation of multiple data sources. Credibility was enhanced by directly observing participants over the duration of the course, which enabled in-session behavior, dialogue, and interactions to be captured when and while they occurred. Credibility was further enhanced as the data from the individual interviews, and debriefing with the facilitators, contributed by elaborating and validating the data recorded during the observations. Data triangulation and involving the user group and interdisciplinary team in the analysis and interpretation of findings contributed to confirmability. Involving the interdisciplinary team in the initial stages of the analysis, and engaging the user group to discuss alternative interpretations of the data, facilitated a critical reflection on how the researchers’ preunderstanding could impact their interpretation of the data. Dependability was enhanced through the consistent intervention format, covering the same topics in both groups and employing the same interview and observational guides for all sessions and interviews. To facilitate the assessment of this study's transferability to other contexts, this article describes the study context, the authors’ preunderstandings, participant characteristics, data collection, and analysis in detail.

Despite the limitations, the findings offer novel and valuable insights into how group interactions, course structure, and facilitation by healthcare professionals can promote coping, healthy behaviors, and empowerment, which warrants further exploration in future studies.

## Conclusion

This study indicates that the combination of group interactions, the course structure, and facilitation by healthcare professionals in a 12-week educational health promotion course can contribute to promoting coping, healthy behaviors, and empowerment in people living with early-stage dementia. Group discussions address knowledge gaps and misconceptions and help adapt information to individual needs. Through a combination of learning, peer support, reminiscing, humor, and facilitator support and validation, participants were empowered to cope with their condition, process negative emotions, and alleviate internalized stigma.

There is a need for more research into group-based educational health promotion interventions for people with early-stage dementia. Future research should focus on determining the optimal timing of these interventions and exploring the characteristics of individuals who benefit the most from attending.

## Supplemental Material

sj-docx-1-son-10.1177_23779608241266686 - Supplemental material for Health Promotion in Early-Stage Dementia: A Focused Ethnographic Study of a 12-Week Group-Based Educational Intervention Supplemental material, sj-docx-1-son-10.1177_23779608241266686 for Health Promotion in Early-Stage Dementia: A Focused Ethnographic Study of a 12-Week Group-Based Educational Intervention by Martine Kajander, Martha Therese Gjestsen, Clive Ballard, Halvor Næss and Ingelin Testad in SAGE Open Nursing

sj-docx-2-son-10.1177_23779608241266686 - Supplemental material for Health Promotion in Early-Stage Dementia: A Focused Ethnographic Study of a 12-Week Group-Based Educational Intervention Supplemental material, sj-docx-2-son-10.1177_23779608241266686 for Health Promotion in Early-Stage Dementia: A Focused Ethnographic Study of a 12-Week Group-Based Educational Intervention by Martine Kajander, Martha Therese Gjestsen, Clive Ballard, Halvor Næss and Ingelin Testad in SAGE Open Nursing

sj-pdf-3-son-10.1177_23779608241266686 - Supplemental material for Health Promotion in Early-Stage Dementia: A Focused Ethnographic Study of a 12-Week Group-Based Educational Intervention Supplemental material, sj-pdf-3-son-10.1177_23779608241266686 for Health Promotion in Early-Stage Dementia: A Focused Ethnographic Study of a 12-Week Group-Based Educational Intervention by Martine Kajander, Martha Therese Gjestsen, Clive Ballard, Halvor Næss and Ingelin Testad in SAGE Open Nursing
