# Supplementary material for: Health Promotion in Early-Stage Dementia: A Focused Ethnographic Study of a 12-Week Group-Based Educational Intervention
Source: SAGE Open Nurs. 2024 Jul 25;10:23779608241266686. doi: 10.1177/23779608241266686 (PMC11273591; doi:10.1177/23779608241266686)
Supplement: sj-docx-1-son-10.1177_23779608241266686 - Supplemental material for Health Promotion in Early-Stage Dementia: A Focused Ethnographic Study of a 12-Week Group-Based Educational Intervention [file sj-docx-1-son-10.1177_23779608241266686.docx]

**Supplemental file:**

**Example of field notes made during a session**

**Observations from session 10: Medications & Talking to Your Health Care Provider - Group B**

Participant 1107 arrive first.

Participant 1106 and Participant 1108 arrives next.

Next, Participant 1105 arrives.

Participant 1104 is the last one to arrive.

Before the session begins, the participants and facilitators discuss a historical figure from the region where the course is being held (details anonymised).

Facilitator 1: welcome!

Repeats the overall aim of the 12-week course: To live as well as possible with dementia

Breathing exercise.

All participants participates in the breathing exercise.

Facilitator 1: the topic of last session was lifelong learning. Participant 1108, you said being open was important to you.

Participant 1108: Mm.

Facilitator 1: [Approaches Participant 1106] Participant 1106, you said that it is important for people to understand what you write and for you to understand what you have written.

Participant 1106: I just have to write it all in capital letters.

Facilitator 1: Participant 1104, it was important to you to be accepted for who you are.

Participant 1104: yes.

Facilitator 1: that was important to you as well, participant 1105.

Facilitator 1: and Participant 1107, you agreed with what the others have said.

Participant 1107: It's not so easy when all the good options have been taken (laughs).

All participants laughs.

Participant 1106: well, that is true.
