# Supplementary material for: Health Promotion in Early-Stage Dementia: A Focused Ethnographic Study of a 12-Week Group-Based Educational Intervention
Source: SAGE Open Nurs. 2024 Jul 25;10:23779608241266686. doi: 10.1177/23779608241266686 (PMC11273591; doi:10.1177/23779608241266686)
Supplement: sj-docx-2-son-10.1177_23779608241266686 - Supplemental material for Health Promotion in Early-Stage Dementia: A Focused Ethnographic Study of a 12-Week Group-Based Educational Intervention [file sj-docx-2-son-10.1177_23779608241266686.docx]

**Interview guide person with dementia**

**Part 1: Introduction**

You have now completed the 12-week health promotion course. How was it, in your experience, to attend this course?

**Part 2: About the health promotion course**

a) Has anything in your life changed as a result of taking this course?

b) How did you find the course material? Was there any information that you were missing?

c) What did you think about the composition of the group?

d) What did you think about the course's length?

e) How did the 12-week health promotion course help you cope with your condition?

f) What did you think about the course facilitators and their role?

**Part 3: Communication**

a) What kind of support do you receive from your family, friends or others close to you?

b) How is the communication between you and your care partner (the person attending the care partner interview)?

c) How do you and your care partner talk about dementia and how the condition affects your life?

d) Have you shared the course booklet with your care partner?

e) Have you and your care partner read the course booklet together and discussed the content? How did you find that?

**Part 4: Final remarks**

Is there anything you would like to add? Things we have not talked about?

Thank you!

**Interview guide care partner**

**Part 1: Introduction**

The person you support has now completed the 12-week health promotion course. Have you any impression of how it has been for him/her to attend this course?

**Part 2: About the health promotion course**

a) Have you noticed any changes in the life of the person you're supporting as a result of taking this course?

b) How did the person you support find the composition of the group?

c) How did the person you support find the course's length?

d) How did the 12-week health promotion course help the person you support cope with his/her condition?

e) How did the person you support find the course facilitators and their role?

**Part 3: Communication**

a) How is the communication between you and the person you support?

b) How do you and the person you support talk about dementia and how the condition affects your life?

c) Have you and the person you support read the course booklet together and discussed the content? How did you find that?

d) How did you find the course material? Was there any information that you were missing?

**Part 4: Final remarks**

Is there anything you would like to add? Things we have not talked about?

Thank you!
